# Serial Ultrasound Assessment of Diaphragmatic Function in Term Neonates During the First 48 Hours of Life

**DOI:** 10.3390/diagnostics16172733

**Published:** 2026-08-26

**Authors:** Bogdan Rotea, Andra Rotea, Vlad Meche, Mădălina Suba, Ovidiu Roșca, Bogdan Cerbu, Ileana Enătescu, Mirabela Dima, Emil Radu Iacob, Adrian Gluhovschi, Daniela Iacob

**Affiliations:** 1 Doctoral School, Victor Babeș University of Medicine and Pharmacy, 300041 Timisoara, Romania; bogdan.rotea@umft.ro (B.R.); andra.alecu@umft.ro (A.R.); bogdan.cerbu@umft.ro (B.C.); 2 Center for Multidisciplinary Research and Management in Pediatric Surgical Conditions, Iosif Nemoianu Street No. 2, 300011 Timisoara, Romania; enatescu.ileana@umft.ro (I.E.); dima.mirabela@umft.ro (M.D.); radueiacob@umft.ro (E.R.I.); iacob.daniela@umft.ro (D.I.); 3 Methodological and Infectious Diseases Research Center, Department of Infectious Diseases, Victor Babeș University of Medicine and Pharmacy, Eftimie Murgu Square No. 2, 300041 Timisoara, Romania; madalina.suba@umft.ro (M.S.); ovidiu.rosca@umft.ro (O.R.); 4 Dr.Victor Babeș Infectious Diseases and Pneumononcology Hospital, 300310 Timisoara, Romania; 5 Department XIII, Discipline of Infectious Diseases, Victor Babeș University of Medicine and Pharmacy, Eftimie Murgu Square No. 2, 300041 Timisoara, Romania; 6 Discipline of Neonatology, Victor Babeș University of Medicine and Pharmacy, 300041 Timisoara, Romania; 7 Department of Pediatric Surgery and Orthopedics, Victor Babeș University of Medicine and Pharmacy, Eftimie Murgu Square No. 2, 300041 Timisoara, Romania; 8 Department XII, Discipline of Obstetrics and Gynecology, Victor Babeș University of Medicine and Pharmacy, 300041 Timisoara, Romania

**Keywords:** diaphragm ultrasound, neonate, respiratory transition, diaphragmatic thickening fraction, lung adaptation

## Abstract

**Background/Objectives**: The early neonatal respiratory transition involves rapid changes in lung aeration and respiratory mechanics, but serial diaphragm-ultrasound trajectories in term neonates remain incompletely characterized. To describe bilateral diaphragmatic thickness, excursion, and diaphragmatic thickening fraction (DTF) during the first 48 h after birth. **Methods**: This prospective, single-center observational study analyzed 112 term neonates with complete ultrasound examinations at 1, 24, and 48 h of life. Bilateral thickness, excursion, DTF, and lung ultrasound score (LUS) were assessed during quiet spontaneous breathing. Serial comparisons used the Friedman test. No a priori sample-size calculation was reported. The analysis included all eligible neonates with complete serial data. **Results**: Right and left excursion increased from 5.65 ± 0.71 and 5.64 ± 0.69 mm at 1 h to 5.91 ± 0.58 and 5.91 ± 0.54 mm at 48 h, respectively (*p* = 0.024 and *p* = 0.033; Kendall’s W = 0.033 and 0.031). These effect sizes were small. DTF remained stable, whereas LUS decreased from 7.01 ± 2.64 at 1 h to 0.09 ± 0.35 at 48 h (*p* < 0.001; Kendall’s W = 0.885). **Conclusions**: In this single-center cohort, lung aeration changed substantially during the first 48 h, whereas diaphragm-ultrasound changes were small. These preliminary trajectories are descriptive and require standardized, multicenter validation before they can be used as reference values or guide clinical decisions.

## 1. Introduction

The transition from fetal to extrauterine life represents a critical period of rapid physiological adaptation, during which the neonatal respiratory system undergoes profound structural and functional changes. At birth, lung aeration replaces fluid-filled alveoli, pulmonary vascular resistance decreases, and effective ventilation must be established within minutes [1]. While lung aeration has traditionally been the primary focus of neonatal respiratory assessment, the role of the respiratory muscles, particularly the diaphragm, has received comparatively less attention in the early postnatal period.

The diaphragm is the principal muscle of respiration in neonates, accounting for the majority of tidal ventilation due to the relative immaturity of accessory respiratory muscles [2]. Its functional capacity is influenced by several factors, including muscle fiber composition, neuromuscular coordination, and mechanical loading conditions. In the immediate postnatal period, the diaphragm must rapidly adapt to increased respiratory demands, changes in lung compliance, and evolving chest wall mechanics. Impaired diaphragmatic function has been associated with respiratory distress, prolonged oxygen requirement, and increased need for respiratory support in neonates [3,4].

Ultrasound has emerged as a non-invasive, bedside tool for assessing diaphragmatic structure and function. Key parameters include diaphragmatic thickness, diaphragmatic excursion, and the diaphragmatic thickening fraction (DTF), which reflects contractile activity during inspiration [5]. In adult and pediatric populations, diaphragm ultrasound has been widely used to evaluate respiratory muscle performance and predict clinical outcomes, particularly in critical care settings. In neonates, however, its application remains relatively limited, and available data are often restricted to small cohorts or single time-point assessments [6].

Recent studies have demonstrated the feasibility of diaphragm ultrasound in term and preterm neonates, suggesting that parameters such as DTF and excursion may provide valuable insights into respiratory adaptation and function [7]. However, the dynamic evolution of these parameters during the first hours and days of life has not been adequately characterized. Most existing studies focus on either pathological populations or isolated measurements, limiting the understanding of normal physiological trajectories in healthy neonates [8,9,10].

A detailed characterization of diaphragmatic function during the early postnatal period may provide important reference data and improve the interpretation of ultrasound findings in both healthy and at-risk neonates. Moreover, serial assessment allows for the evaluation of temporal patterns, which may be more informative than single measurements when assessing respiratory adaptation.

Therefore, the aim of this study was to perform a serial ultrasound evaluation of diaphragmatic function in term neonates during the first 48 h of life, focusing on diaphragmatic thickness, excursion, and thickening fraction. We hypothesized that these parameters undergo measurable changes during early postnatal adaptation and that their longitudinal assessment can contribute to a better understanding of neonatal respiratory physiology.

## 2. Materials and Methods

### 2.1. Study Design and Setting

This prospective, observational, longitudinal study was conducted in a tertiary-level maternity unit (Neonatology Department of the “Louis Turcanu” Children’s Hospital in Timisoara, Romania). Reporting was structured in accordance with the STROBE recommendations for observational studies. The study was exploratory: no a priori sample-size calculation was performed, and all eligible neonates with complete serial ultrasound data were included. A post hoc power calculation was not used because it would be determined by the observed *p*-value and would not provide independent information beyond the reported effect sizes and confidence intervals.

### 2.2. Study Population

#### 2.2.1. Inclusion Criteria

Term neonates (gestational age ≥ 37 weeks) were eligible for inclusion if they:

- Were clinically stable after birth;
- Were evaluated within the first hour of life;
- Had parental consent for participation.

#### 2.2.2. Exclusion Criteria

Neonates were excluded if they presented:

- Major congenital anomalies;
- Congenital diaphragmatic or pulmonary malformations;
- Need for invasive respiratory support at birth;
- Significant perinatal asphyxia.

Cesarean delivery and transient supplemental oxygen during the first postnatal hour were not exclusion criteria. Neonates requiring invasive respiratory support were excluded. Maternal diabetes, non-invasive respiratory support, clinically diagnosed respiratory distress, congenital heart disease, infection, feeding status, sedative exposure, and other perinatal complications were also excluded.

### 2.3. Clinical Data Collection

Baseline demographic and clinical data were recorded for all neonates, including:

- Sex;
- Gestational age (weeks);
- Birth weight (g);
- Length and anthropometric parameters;
- Apgar score at 1 and 5 min;
- Mode of delivery (vaginal or cesarean section).

During follow-up, the need for oxygen therapy was recorded as a binary variable (0 = no, 1 = yes).

### 2.4. Ultrasound Protocol and Timing of Assessments

Ultrasound evaluations were performed at three predefined time points:

- At 1 h of life (target window: 60 ± 15 min);
- At 24 h of life;
- At 48 h of life.

All examinations were performed according to a standardized acquisition protocol by one sonographer. The primary operator had 5 years of neonatal and point-of-care ultrasound experience and had completed 5 years of fellowship. Observers were blinded to prior ultrasound measurements and relevant clinical data.

### 2.5. Diaphragm Ultrasound

Diaphragm ultrasound was performed using a LOGIQ E10 (GE Healthcare) system with a high-frequency linear transducer operating at 12 MHz. Neonates were examined supine or semi-recumbent during quiet spontaneous breathing. Feeding status and behavioral/sleep state at the time of scanning were not recorded.

For thickness measurements, the linear transducer was applied to the lateral thoracic wall at the mid-axillary line, between the eighth and tenth intercostal spaces, within the zone of apposition. Here, “lateral” refers to the position of the probe on the lateral chest wall rather than to the direction of the ultrasound beam. The diaphragm was identified as the characteristic three-layer structure between the pleural and peritoneal interfaces. Thickness was measured perpendicular to the muscle fibers at end-expiration and end-inspiration. For excursion, a curvilinear probe of the same device was placed using a subcostal approach, and the M-mode cursor was aligned as perpendicular as possible to diaphragmatic motion, at a frequency of 3 MHz. Excursion was measured from the end-expiratory baseline to the peak inspiratory displacement. All measurements were obtained during quiet spontaneous breathing, avoiding crying and gross body movement.

- Diaphragmatic thickness at end-expiration (mm);
- Diaphragmatic thickness at end-inspiration (mm);
- Diaphragmatic excursion (mm) assessed using M-mode.

The diaphragmatic thickening fraction (*DTF*) was calculated using the formula:

$$DTF(\%)=\frac{Thickness_{inspiration}-Thickness_{expiration}}{Thickness_{expiration}}\times 100$$

Right and left hemidiaphragm measurements were retained and analyzed separately rather than averaged. Each reported value represented the mean of 3 technically acceptable respiratory cycles. Intra-observer and inter-observer reproducibility were not assessed (Figure S1).

### 2.6. Lung Ultrasound (Secondary Data)

Lung ultrasound was performed using a six-zone protocol:

- Right anterior superior;
- Right anterior inferior;
- Right lateral;
- Left anterior superior;
- Left anterior inferior;
- Left lateral.

Each zone was scored from 0 to 3, and a total lung ultrasound score (LUS) was automatically calculated.

At 24 and 48 h, lung ultrasound images were saved only when pathological findings were identified.

### 2.7. Data Management

Serial measurements were recorded for each neonate at all three time points. The primary longitudinal analysis used a complete-case cohort of 112 neonates. Right and left measurements were analyzed separately.

### 2.8. Statistical Analysis

Statistical analysis was performed using Python 3.14.6. Continuous variables were summarized as mean ± standard deviation with 95% confidence intervals or as median (interquartile range), according to their distribution. Distributional assumptions were assessed using Shapiro–Wilk test and visual Q–Q plots. Serial diaphragm measurements and LUS were analyzed using the Friedman test because of the observed non-normality. Kendall’s W was reported as the omnibus effect size. When the omnibus test was significant, pairwise time-point comparisons were performed using a paired test with Bonferroni adjustment; adjusted *p*-values and paired effect estimates with 95% confidence intervals should be reported. Right-versus-left comparisons used a paired *t*-test. Correlations used Pearson or Spearman coefficients as appropriate. The principal analysis was complete-case: no imputation was performed. Two-sided adjusted *p* < 0.05 was considered statistically significant.

## 3. Results

### 3.1. Baseline Characteristics

A total of 121 neonates were initially recorded in the database. After applying predefined inclusion criteria for term gestation (37–42 weeks) and excluding cases without complete serial ultrasound data, the final analysis cohort included 112 term neonates. This cohort represents a homogeneous population suitable for evaluating physiological respiratory adaptation.

**Figure 1:**
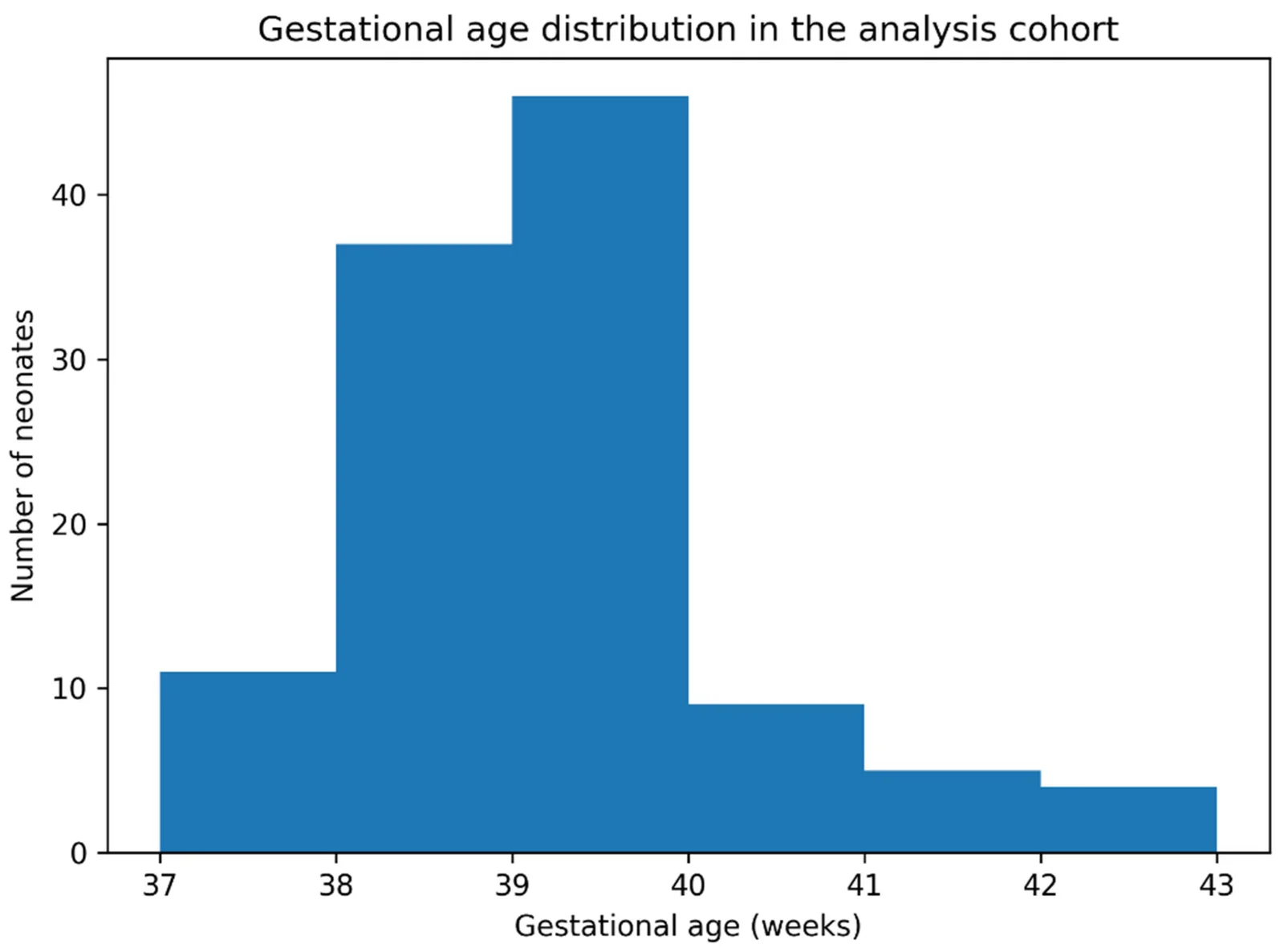
Gestational age distribution.

The study population included 60 male neonates (53.6%) and 52 female neonates (46.4%), with no significant sex imbalance. The mean gestational age was 38.70 ± 1.12 weeks, with a median of 39 weeks, confirming that the cohort was centered around full-term maturity (Figure 1).

Distribution of gestational age (weeks) in the study cohort. The histogram demonstrates a concentration of cases around full-term gestation, with a mean gestational age of 38.7 weeks.

**Figure 2:**
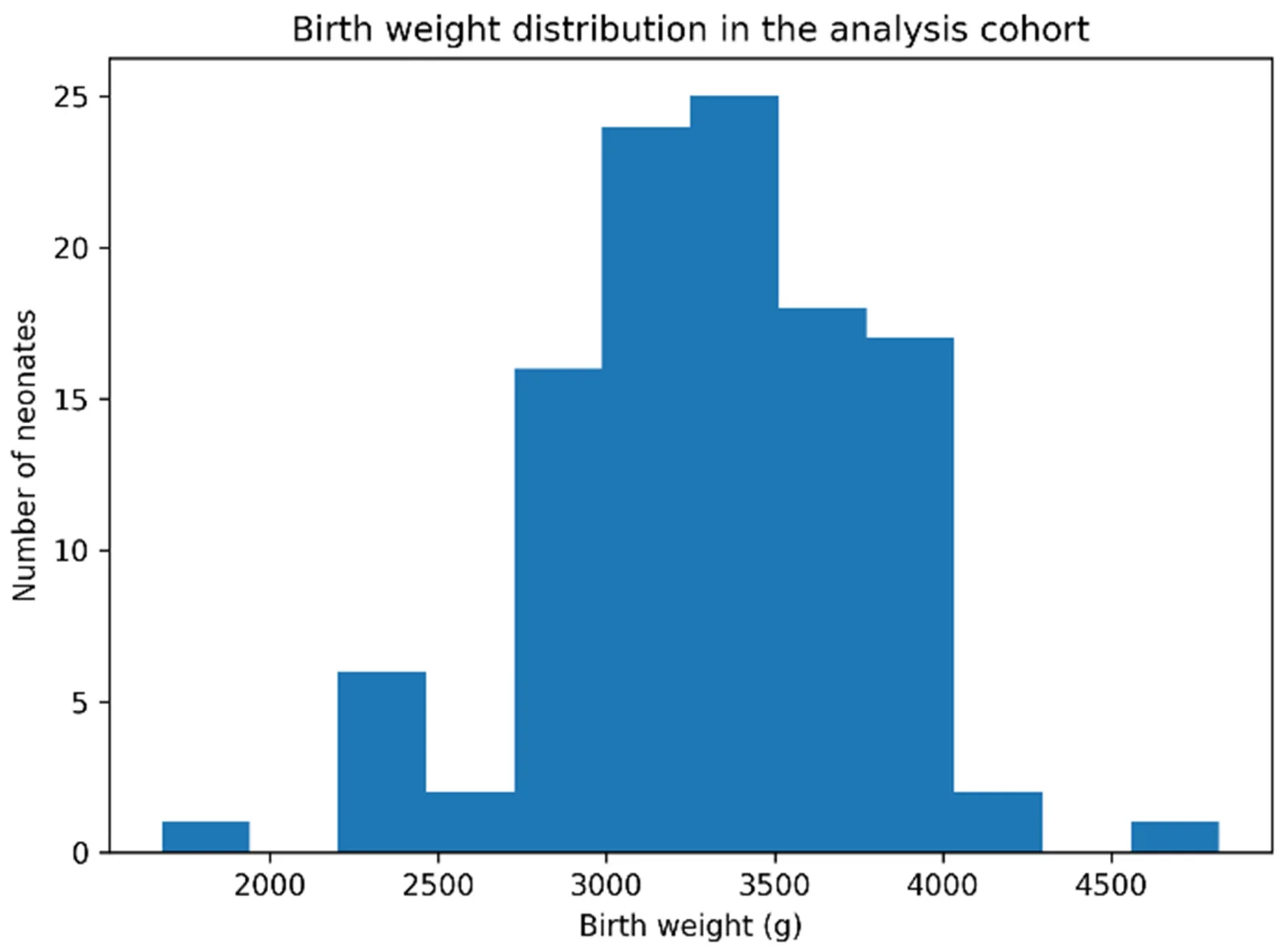
Birth weight distribution.

Birth weight averaged 3295.89 ± 473.01 g, with a relatively narrow distribution, indicating an absence of significant growth restriction or macrosomia (Figure 2). Anthropometric parameters, including length and cranial circumference, were within expected physiological ranges for term neonates.

Distribution of birth weight (grams) in the study cohort. Values are centered around normal birth weight ranges for term neonates, with limited variability.

Regarding delivery mode, 88 neonates (78.6%) were delivered by cesarean section, while 23 neonates (20.5%) were delivered vaginally; one case had missing data. This distribution reflects a cesarean-predominant cohort, which is relevant given the known association between cesarean delivery and delayed lung fluid clearance.

At 1 h of life, 24 neonates (21.4%) required oxygen therapy, while the remaining 78.6% maintained adequate oxygenation without support. Importantly, no neonate required oxygen therapy at 24 or 48 h, indicating rapid clinical stabilization across the cohort (Table 1).

These findings confirm that the study population consisted predominantly of clinically stable term neonates undergoing physiological respiratory transition, providing a robust framework for interpreting serial ultrasound measurements.

### 3.2. Evolution of Diaphragmatic Thickness

Diaphragmatic thickness demonstrated modest but statistically measurable temporal variation, particularly at the level of the right hemidiaphragm.

#### 3.2.1. Right Hemidiaphragm

Right expiratory thickness showed a statistically significant variation over time:

- 1 h: 2.25 ± 0.27 mm;
- 24 h: 2.18 ± 0.25 mm;
- 48 h: 2.26 ± 0.30 mm.

(Friedman χ^2^ = 6.43, *p* = 0.040, Kendall’s W = 0.029)

The temporal pattern consisted of:

- A decrease from 1 h to 24 h;
- Followed by recovery at 48 h.

Right inspiratory thickness did not show statistically significant variation:

- 1 h: 2.72 ± 0.28 mm;
- 24 h: 2.66 ± 0.30 mm;
- 48 h: 2.72 ± 0.35 mm (*p* = 0.206).

#### 3.2.2. Left Hemidiaphragm

Left-sided thickness measurements were stable across all time points. Left expiratory thickness was 2.24 mm at 1 h, 2.2 mm at 24 h, and 2.24 mm at 48 h (omnibus *p* = 0.304); left inspiratory thickness was 2.7 mm, 2.64 mm, and 2.7 mm, respectively (omnibus *p* = 0.497).

- Left expiratory thickness: *p* = 0.304;
- Left inspiratory thickness: *p* = 0.497.

Overall, these findings indicate that diaphragmatic thickness undergoes minimal structural change during the first 48 h, with only subtle transient variation observed on the right side.

**Figure 3:**
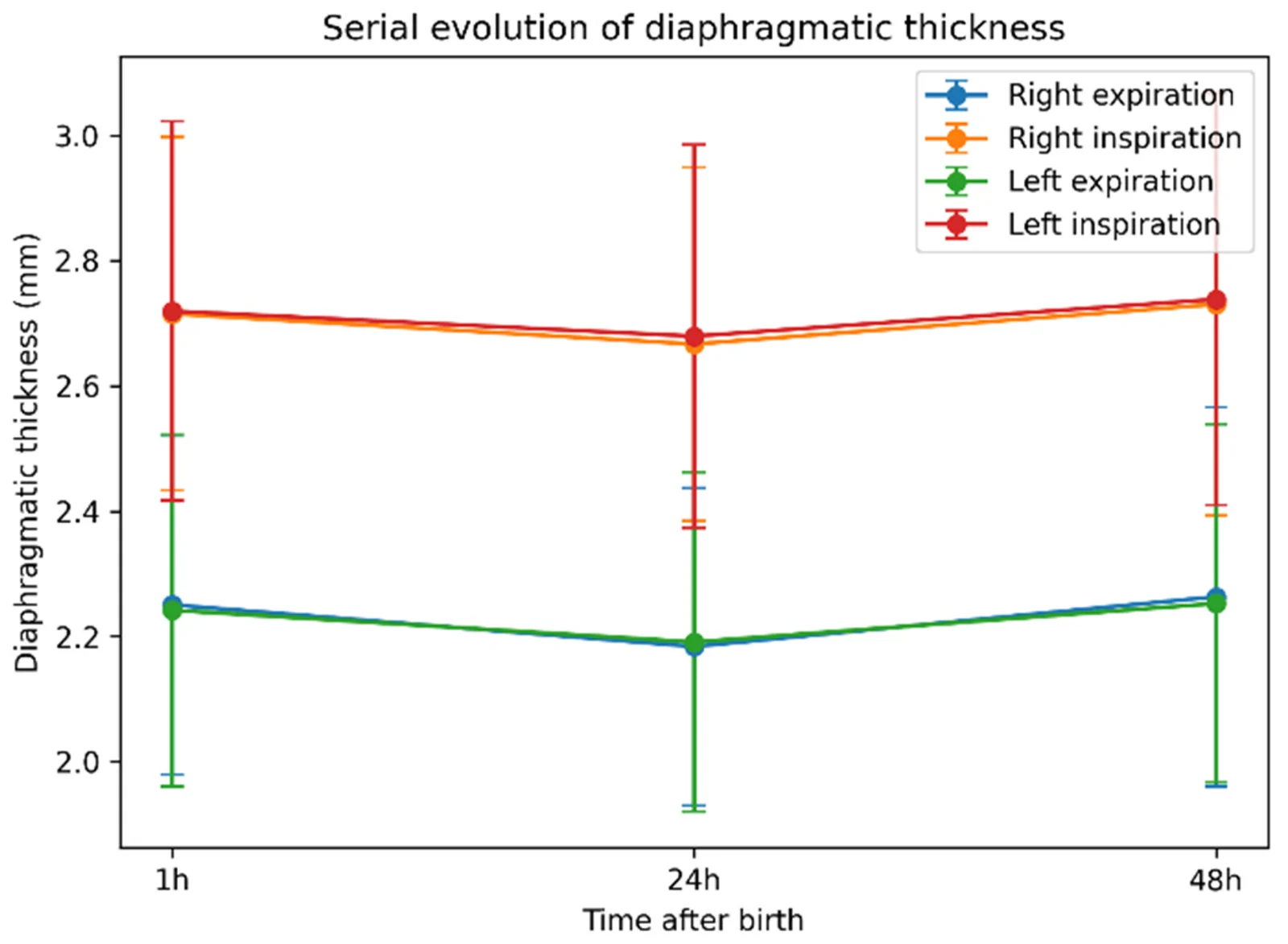
Serial evolution of diaphragmatic thickness. Mean ± SD thickness at end-expiration and end-inspiration for each hemidiaphragm at 1, 24, and 48 h. Right expiratory thickness varied over time (Friedman χ^2^ = 6.43, *p* = 0.040; Kendall’s W = 0.029), whereas right inspiratory and left-sided thickness measures did not show significant temporal variation.

The low effect size (Kendall’s W ≈ 0.03) suggests that, although statistically significant, the magnitude of change is small, reinforcing the interpretation of early structural stability with minor adaptive fluctuation (Figure 3).

Mean diaphragmatic thickness (mm) measured at end-expiration and end-inspiration for both hemidiaphragms at 1 h, 24 h, and 48 h of life. Minor variations are observed over time, with no consistent directional trend, suggesting structural stability of the diaphragm during early adaptation.

### 3.3. Diaphragmatic Excursion

Diaphragmatic excursion demonstrated the clearest and most consistent temporal trend among all diaphragmatic parameters.

**Right hemidiaphragm**

- 1 h: 5.65 ± 0.71 mm;
- 24 h: 5.80 ± 0.62 mm;
- 48 h: 5.91 ± 0.58 mm.

(Friedman χ^2^ = 7.47, *p* = 0.024, Kendall’s W = 0.033)

**Left hemidiaphragm**

- 1 h: 5.64 ± 0.69 mm;
- 24 h: 5.80 ± 0.62 mm;
- 48 h: 5.91 ± 0.54 mm.

(Friedman χ^2^ = 6.83, *p* = 0.033, Kendall’s W = 0.031)

**Temporal pattern**

Both hemidiaphragms exhibited a progressive increase in excursion, with the most pronounced difference between 1 h and 48 h.

**Variability**

**Figure 4:**
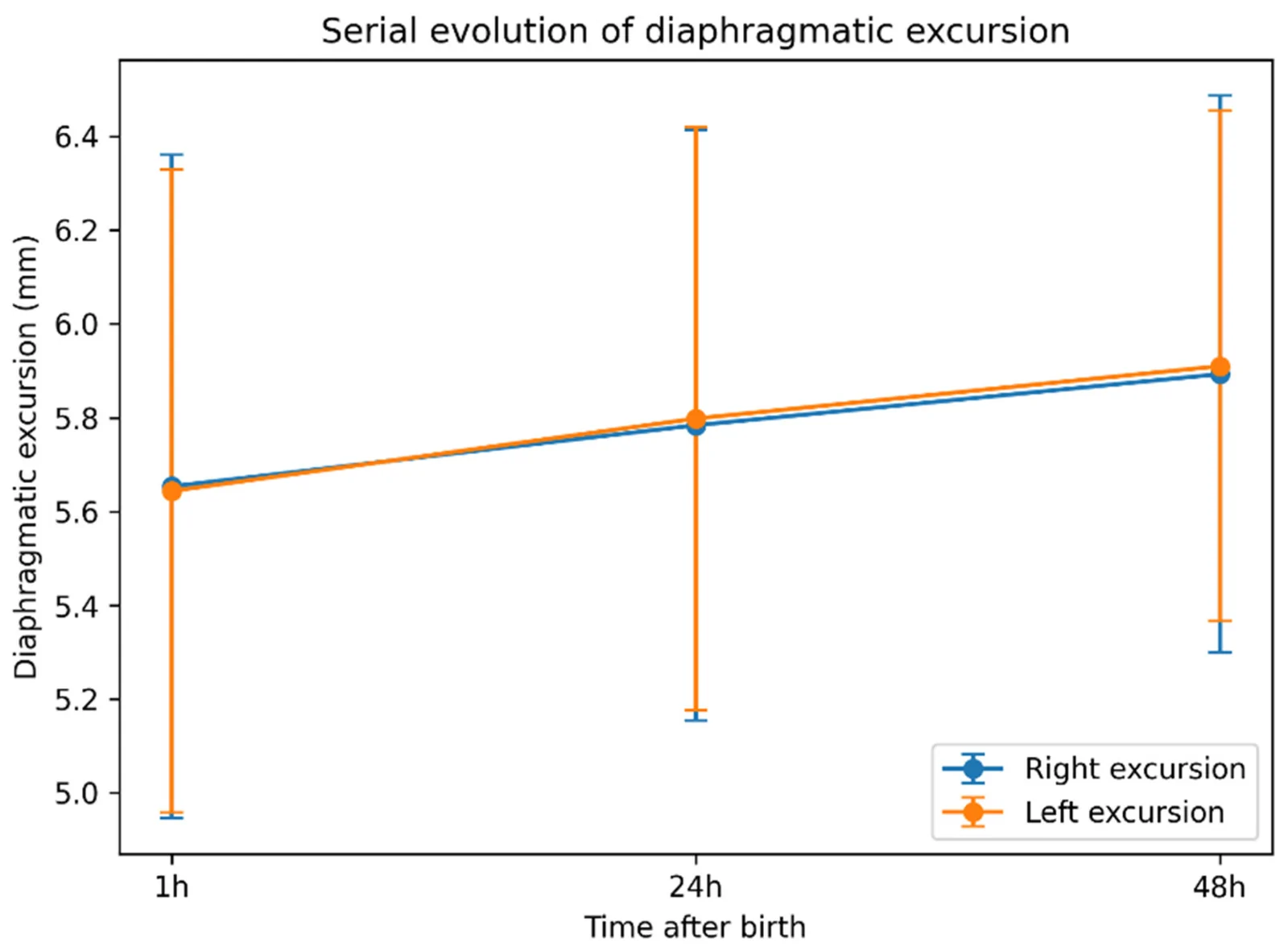
Serial evolution of diaphragmatic excursion. Mean ± SD excursion at 1, 24, and 48 h. Right: Friedman χ^2^ = 7.47, *p* = 0.024, Kendall’s W = 0.033. Left: Friedman χ^2^ = 6.83, *p* = 0.033, Kendall’s W = 0.031. Both effect sizes were small.

The bilateral increase in mean excursion was approximately 0.26–0.27 mm (about 4.6–4.8%) over 48 h. Although statistically significant, the corresponding Kendall’s W values (0.031–0.033) indicate a small effect. The result therefore describes a modest cohort-level temporal shift and does not establish a threshold for clinical decision-making (Figure 4).

Serial measurements of right and left diaphragmatic excursion (mm) at 1 h, 24 h, and 48 h of life. Both hemidiaphragms demonstrate a progressive increase in excursion over time, reflecting improving mechanical efficiency of diaphragmatic function during early postnatal adaptation.

### 3.4. Diaphragmatic Thickening Fraction (DTF)

DTF remained remarkably stable across all time points, with no statistically significant changes.

**Right DTF**

- 1 h: 20.96 ± 3.93%;
- 24 h: 22.05 ± 4.99%;
- 48 h: 20.93 ± 3.78% (*p* = 0.065).

**Left DTF**

- 1 h: 21.52 ± 3.97%;
- 24 h: 22.51 ± 4.75%;
- 48 h: 21.72 ± 3.79% (*p* = 0.252).

Although a slight increase was observed at 24 h, particularly on the right side, these changes did not reach statistical significance (Table 2).

**Figure 5:**
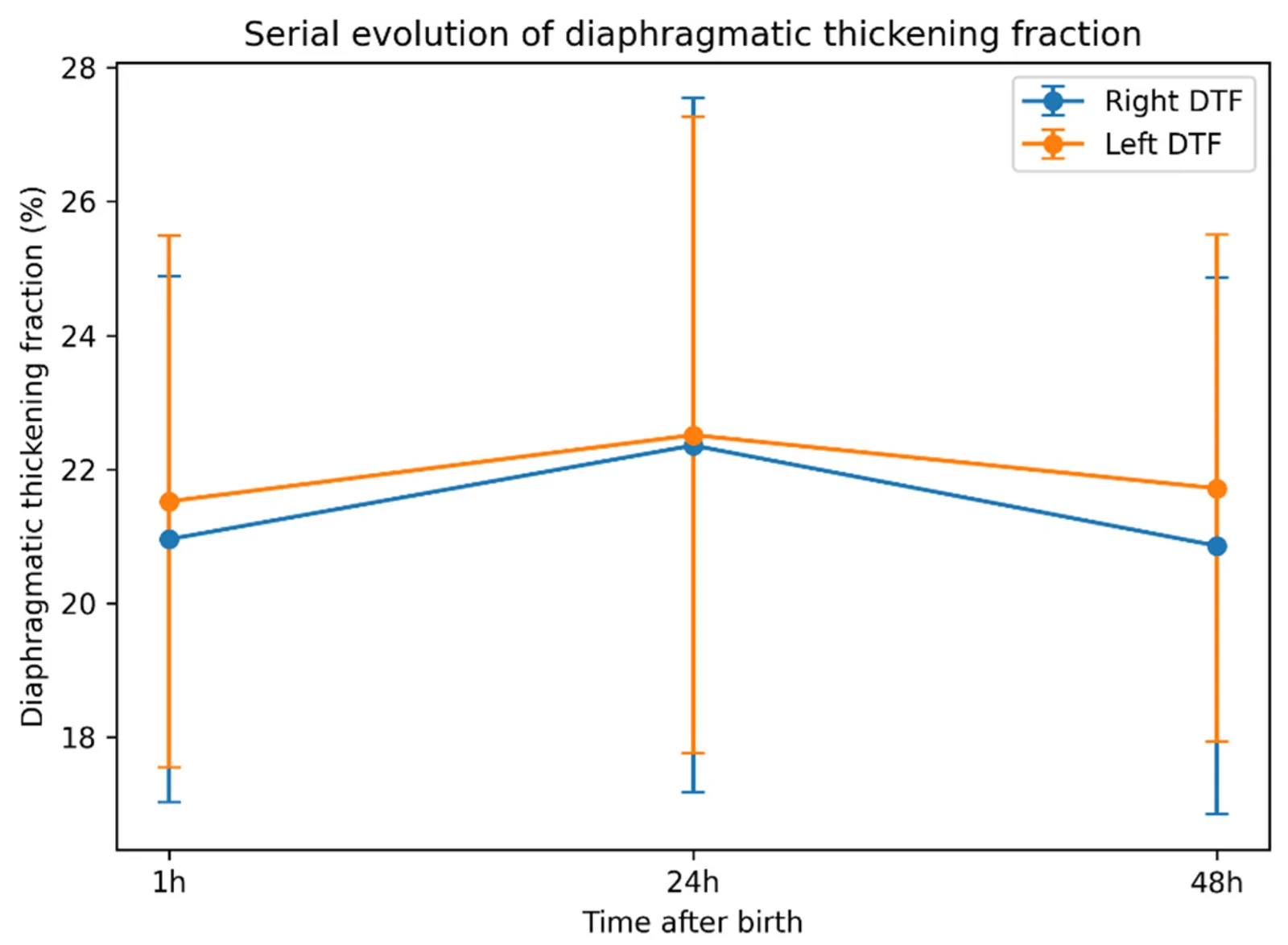
Evolution of diaphragmatic thickening fraction (DTF). Mean ± SD bilateral DTF at 1, 24, and 48 h. No significant temporal variation was detected (right *p* = 0.065; left *p* = 0.252).

The absence of significant temporal variation indicates that diaphragmatic contractile function is established early and remains stable, in contrast to the progressive changes observed in excursion (Figure 5).

Serial measurements of right and left diaphragmatic thickening fraction (%) at 1 h, 24 h, and 48 h of life. DTF values remain relatively stable across all time points, indicating preserved diaphragmatic contractile function during the neonatal transition period.

### 3.5. Right vs. Left Hemidiaphragm

Bilateral comparisons demonstrated a high degree of symmetry across most parameters.

No statistically significant differences were observed between the right and left hemidiaphragm for expiratory thickness, inspiratory thickness or excursion at any time point.

The only statistically significant difference was observed for DTF at 48 h:

- Right: 20.93 ± 3.78%;
- Left: 21.72 ± 3.79% (*p* = 0.017).

However, the absolute difference was small (<1%), suggesting limited clinical relevance.

Overall, the observed bilateral differences were small in this cohort. However, the data do not establish that either hemidiaphragm can be used interchangeably in clinical practice, particularly because left-sided acquisition may be technically more difficult and reproducibility was not quantified.

### 3.6. Lung Ultrasound Findings

Lung ultrasound demonstrated the most pronounced temporal change among all evaluated parameters.

**Total LUS**

- 1 h: 7.01 ± 2.64;
- 24 h: 1.20 ± 1.46;
- 48 h: 0.09 ± 0.35.

(Friedman χ^2^ = 198.14, *p* < 0.001, Kendall’s W = 0.885)

**Temporal pattern**

- Sharp decrease from 1 h to 24 h;
- Near-complete normalization by 48 h.

**Figure 6:**
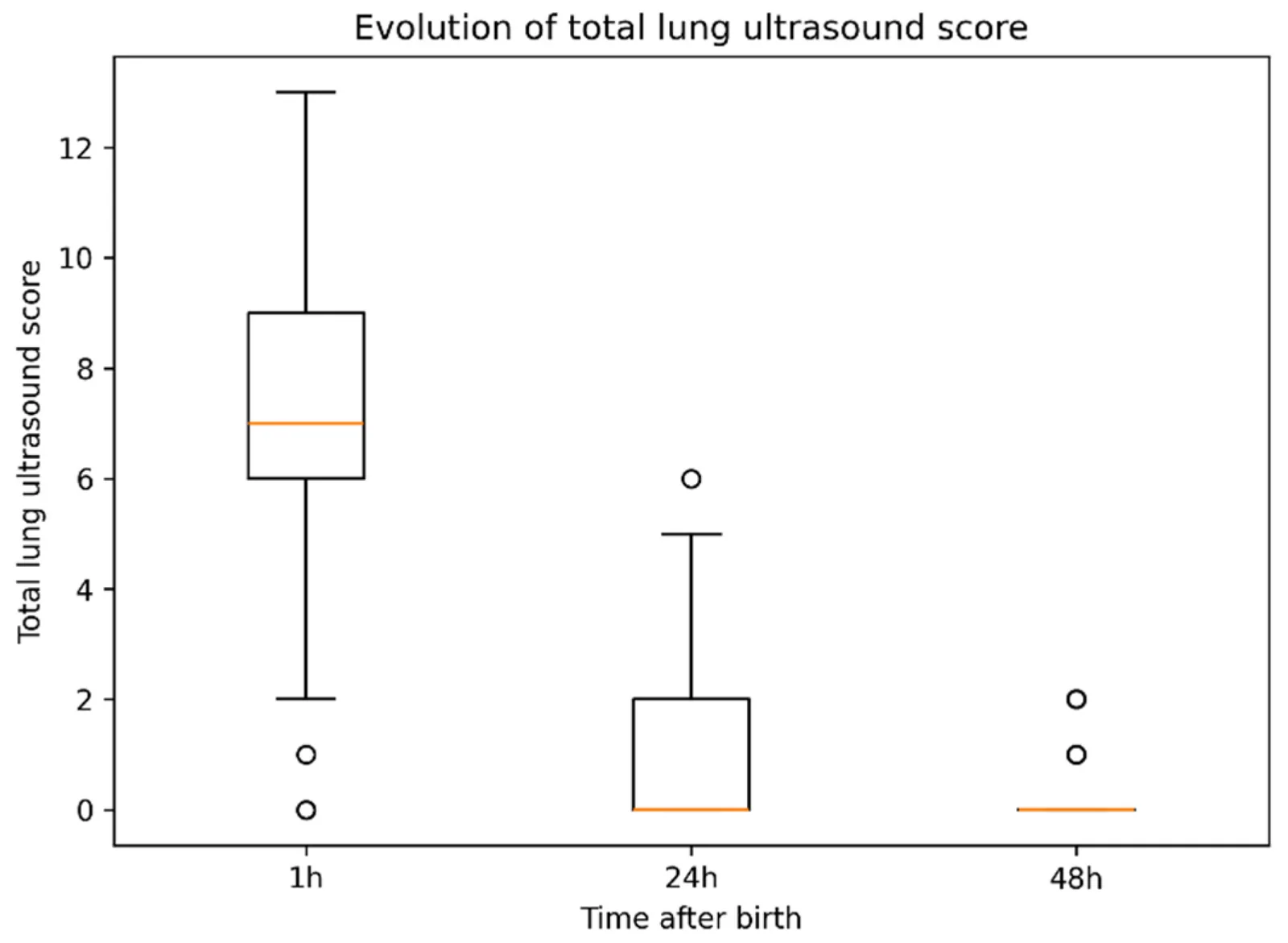
Evolution of lung ultrasound score (LUS). LUS at 1, 24, and 48 h decreased markedly over time (Friedman χ^2^ = 198.14, *p* < 0.001; Kendall’s W = 0.885).

The very large effect size (Kendall’s W = 0.885) indicates that time explains most of the variation in LUS, making it the strongest temporal marker in the dataset (Figure 6).

Boxplot representation of total lung ultrasound score at 1 h, 24 h, and 48 h of life. A marked decrease in LUS is observed over time, reflecting rapid lung aeration and clearance of residual lung fluid during the first 48 h of life.

### 3.7. Oxygen Therapy and Baseline Severity

At 1 h of life:

- Oxygen group: median LUS 10.0;
- No oxygen group: median LUS 7.0 (*p* < 0.001).

**Figure 7:**
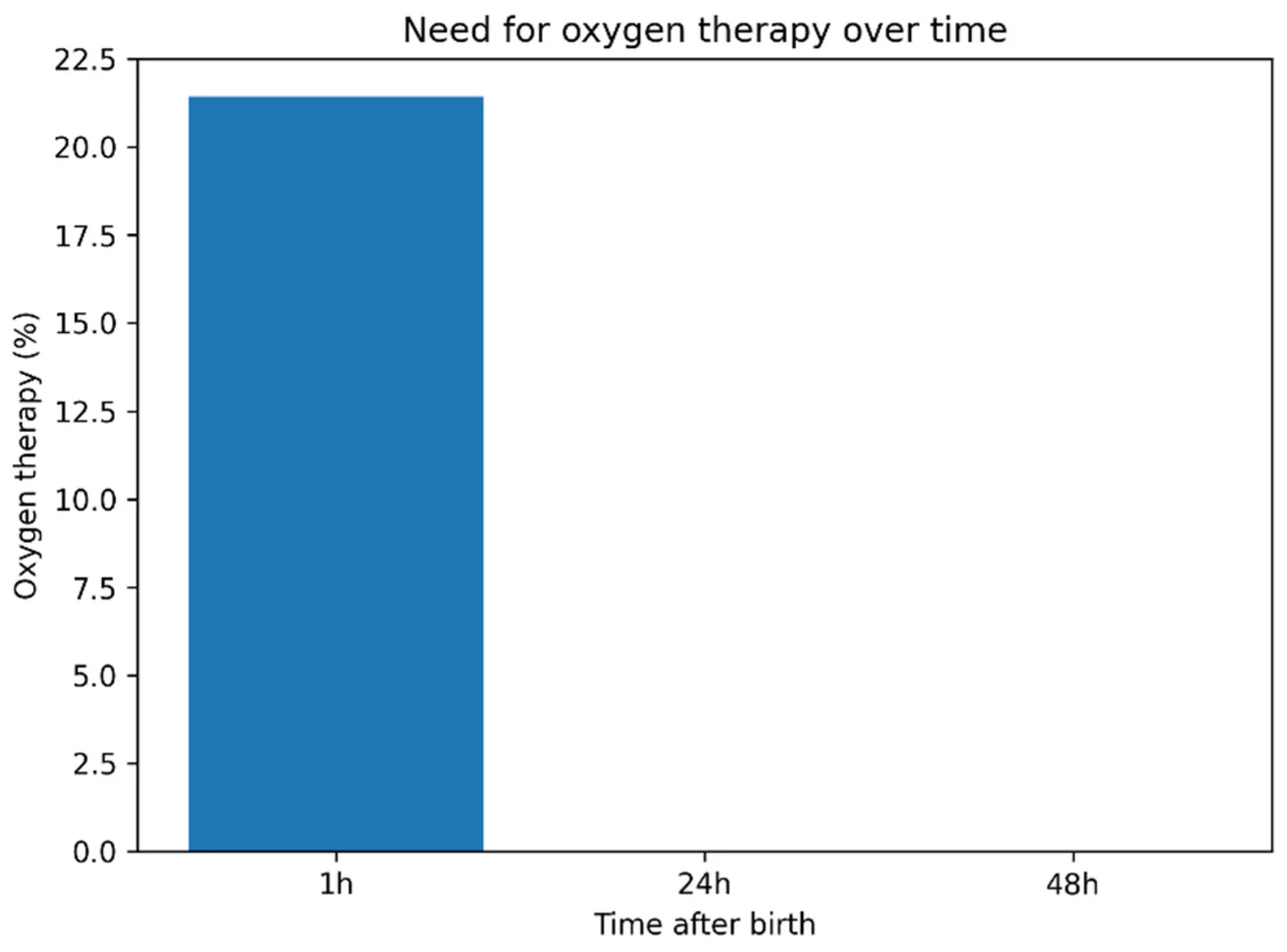
Oxygen therapy requirement over time. Twenty-four of 112 neonates (21.4%) received supplemental oxygen at 1 h; none received oxygen at 24 or 48 h.

No neonate required oxygen therapy at 24 or 48 h (Figure 7).

Proportion of neonates requiring oxygen therapy at 1 h, 24 h, and 48 h of life. Oxygen therapy was required in a subset of neonates at 1 h but was no longer needed at later time points, indicating rapid clinical stabilization.

No significant differences were observed for:

- Diaphragmatic excursion;
- DTF.

**Figure 8:**
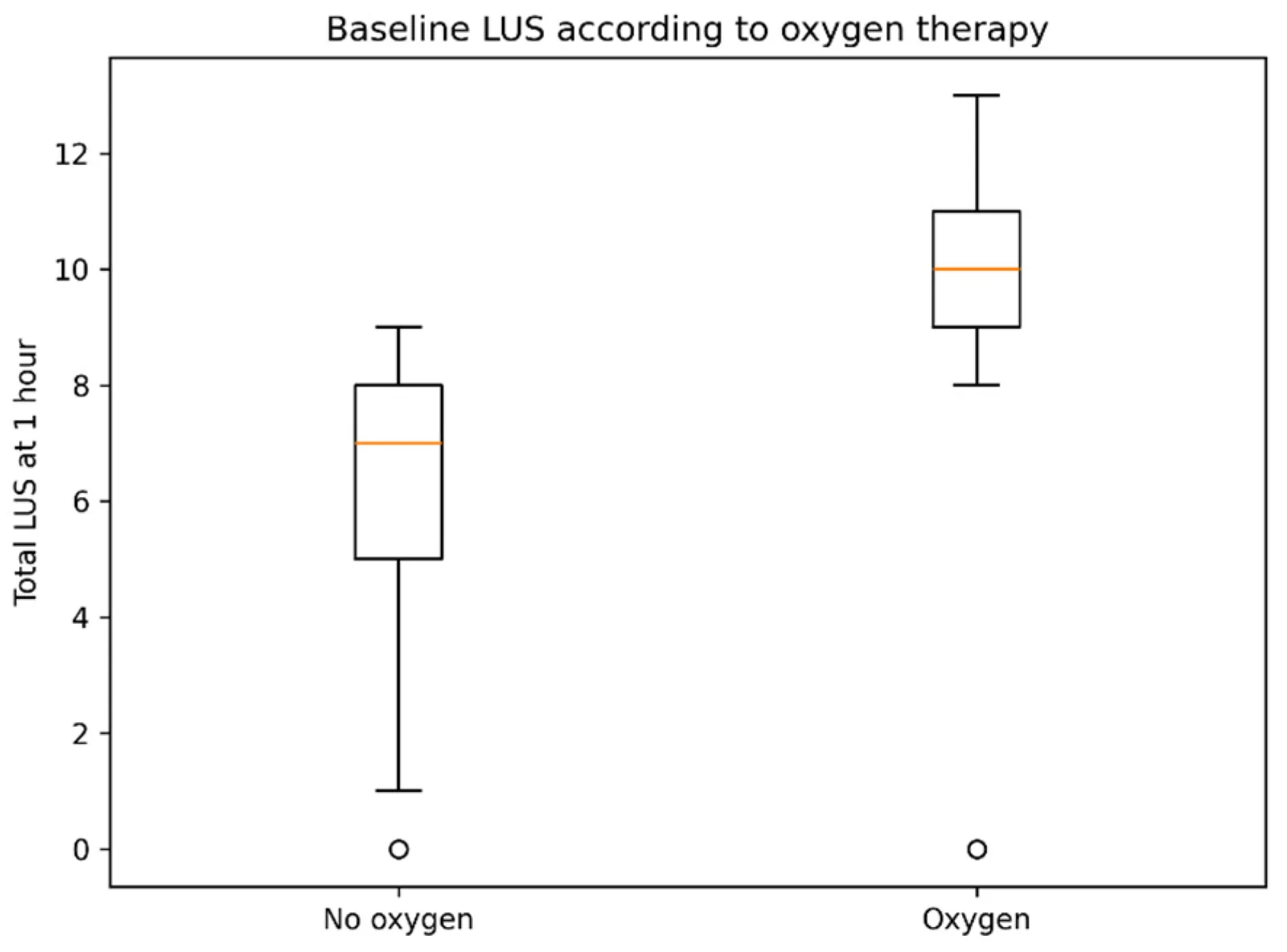
Lung ultrasound score (LUS) at 1 h according to oxygen-therapy status. Median LUS was 10.0 among neonates receiving oxygen and 7.0 among those not receiving oxygen (*p* < 0.001).

This indicates that early oxygen requirement is associated with impaired lung aeration but not with measurable diaphragmatic dysfunction (Figure 8).

Comparison of total lung ultrasound score at 1 h between neonates requiring oxygen therapy and those not requiring support. Higher LUS values are observed in the oxygen therapy group, indicating reduced lung aeration in these neonates.

### 3.8. Correlation Analysis

No statistically significant correlations were identified between:

- LUS and diaphragmatic excursion;
- LUS and DTF;

At any time point.

Although both lung aeration and diaphragmatic function improved over time, the absence of correlation suggests that these processes evolve in parallel but are not linearly dependent at the individual level.

### 3.9. Integrated Temporal Pattern

Combining all parameters reveals a coherent physiological sequence:

At 1 h: High LUS, lower excursion, and greater variability;

At 24 h: Rapid improvement in LUS, an increasing excursion, with stabilization of thickness;

At 48 h: Near-normal LUS, maximal excursion, with minimal variability.

## 4. Discussion

### 4.1. Principal Findings

This study describes serial changes in lung aeration and diaphragm-ultrasound measurements during the first 48 h after birth. The large decline in LUS was the dominant temporal finding (Kendall’s W = 0.885). In comparison, changes in diaphragm thickness and excursion were small (W approximately 0.03), and DTF remained stable. Statistical significance should therefore not be equated with clinical importance: the diaphragm findings represent modest group-level shifts and cannot currently define abnormality or guide individual management.

The marked reduction in LUS between 1 and 48 h represents the strongest signal in our dataset, with a very large effect size (Kendall’s W = 0.885), indicating that lung aeration is the dominant component of early respiratory adaptation. This finding is consistent with previous studies demonstrating that lung ultrasound can capture rapid postnatal clearance of fetal lung fluid and transition toward air-filled lungs [11].

In contrast to the large LUS change, diaphragm-ultrasound measurements changed only modestly. The excursion findings are compatible with evolving respiratory mechanics, but the small effect sizes and absence of respiratory-effort measurements preclude stronger physiological or clinical conclusions.

DTF remained stable at the three assessment points. This observation does not establish that neonatal diaphragm contractility was fully mature or unchanged, because DTF depends on acquisition technique, breathing pattern, respiratory effort, and measurement reproducibility [12,13]. 

### 4.2. Lung Aeration as the Primary Driver of Early Adaptation

The rapid decrease in LUS observed in our cohort, from a mean value of 7.01 at 1 h to near-zero at 48 h, reflects the well-described physiological process of lung fluid clearance and alveolar recruitment after birth.

Lung ultrasound has been extensively validated as a tool for assessing neonatal lung aeration, with several studies demonstrating its ability to track the transition from fluid-filled to air-filled lungs [14,15]. Raimondi et al. showed that LUS decreases rapidly in the first 24 h of life and correlates with oxygenation status [16], while Liu et al. demonstrated its diagnostic value in transient tachypnea of the newborn (TTN) [17].

The magnitude of LUS change in our study is particularly striking and suggests that, even in a cohort of term neonates without significant pathology, lung aeration undergoes rapid and near-complete normalization within the first 48 h. This supports previous observations that lung adaptation is the dominant physiological event in early neonatal respiratory transition [18].

Higher LUS at 1 h was observed among neonates receiving supplemental oxygen. Because oxygen administration was not randomized and may reflect clinician thresholds and other clinical features, this association does not establish LUS as the primary determinant of respiratory-support need. It is nevertheless consistent with prior studies evaluating LUS in early neonatal respiratory disease [19].

### 4.3. Diaphragmatic Excursion as a Marker of Functional Adaptation

Diaphragmatic excursion demonstrated a statistically significant increase over time, representing the most consistent functional change among diaphragmatic parameters.

Unlike LUS, which reflects lung aeration, diaphragmatic excursion reflects the mechanical displacement of the diaphragm during inspiration and is influenced by both muscle function and respiratory system compliance [20].

The mean increase in excursion was only 0.26–0.27 mm over 48 h. Several mechanisms could contribute to this modest change. Clearance of fetal lung fluid, recruitment of aerated lung volume, falling pulmonary vascular resistance, and evolving functional residual capacity may alter respiratory-system compliance and the mechanical load against which the diaphragm contracts [1,3,5,7,12]. Changes in chest-wall configuration and breathing pattern may also affect M-mode displacement independently of intrinsic muscle strength [21]. Because DTF did not change and individual LUS and excursion were not correlated, the present data cannot establish that improved aeration caused the increase in excursion. The result is more appropriately interpreted as compatible with evolving respiratory mechanics.

Prior studies in mechanically ventilated or preterm populations have examined excursion in relation to respiratory performance and outcomes [22,23]. These findings cannot be transferred directly to stable term neonates, and the present study was not designed to evaluate prediction or diagnostic accuracy.

Accordingly, the small increase observed here should be treated as a descriptive trajectory rather than evidence of clinically important improvement in mechanical efficiency.

### 4.4. Stability of Diaphragmatic Contractility (DTF)

In contrast to diaphragmatic excursion, DTF remained stable across all time points, with no statistically significant changes.

Stable DTF values indicate that no temporal change was detected with this protocol and sample; they do not prove that contractile function was already established or invariant after birth.

The values can be compared with prior neonatal ultrasound studies [24,25], but reproducibility and agreement cannot be inferred from similarity between cohort means.

The combination of stable DTF and slightly increasing excursion may indicate that a broadly similar relative thickening pattern produced marginally greater displacement as postnatal mechanics evolved. However, DTF is sensitive to image plane, end-inspiratory and end-expiratory timing, breathing variability, and operator technique [26,27]. Without reproducibility estimates or simultaneous measures of respiratory effort, stable DTF cannot be taken as proof that contractile function was fully mature or unchanged.

### 4.5. Bilateral Symmetry of Diaphragmatic Function

Our results demonstrated no clinically significant differences between the right and left hemidiaphragm for most parameters, with only a small difference in DTF at 48 h. Bilateral symmetry of diaphragmatic function is expected in healthy neonates, as both hemidiaphragms contribute equally to ventilation under normal conditions [28].

The descriptive bilateral similarity is reassuring, but without formal intra- and interobserver analyses, it cannot be used as evidence of measurement reliability.

The absence of marked asymmetry is compatible with a clinically stable cohort, but it does not by itself distinguish physiological from pathological adaptation.

### 4.6. Relationship Between Lung Aeration and Diaphragmatic Function

LUS decreased and excursion increased over time, but no significant cross-sectional correlations were detected. This does not establish independence or coordinated improvement; the analyses may be influenced by range restriction, nonlinear relationships, timing, and measurement error.

The findings therefore support only the descriptive conclusion that the two ultrasound domains showed different cohort-level trajectories.

LUS primarily reflects parenchymal aeration, while diaphragmatic parameters reflect muscle function and mechanical performance [29]. Therefore, the absence of correlation does not indicate independence, but rather highlights the complementary nature of these measurements.

The absence of a cross-sectional correlation should be interpreted cautiously. LUS and diaphragm ultrasound describe different physiological domains—parenchymal aeration and respiratory-muscle geometry/motion—and both are affected by timing, respiratory effort, body position, and measurement error. Their complementary information is a testable hypothesis rather than evidence that combining them improves diagnosis or outcomes.

### 4.7. Clinical Implications

The present findings do not establish a clinical decision threshold or demonstrate that serial diaphragm ultrasound changes management. LUS showed a large temporal change and was higher among neonates receiving oxygen at 1 h, whereas the diaphragm changes were small and did not discriminate oxygen requirement. At this stage, the results are best viewed as descriptive trajectories that may inform the design of future reference studies and hypothesis-driven clinical investigations.

Lung ultrasound has already been shown to be useful for diagnosing and monitoring neonatal respiratory disorders [30,31,32], while diaphragm ultrasound is emerging as a tool for assessing respiratory muscle function [33,34,35,36].

Within this cohort, LUS was the most strongly time-varying measure, excursion showed a small temporal change, and DTF showed no detectable temporal change. These observations are descriptive and should not be used to classify individual neonates.

### 4.8. Limitations

This study has several limitations. It was conducted at a single center and included a selected cohort of stable term neonates, limiting generalizability to preterm, critically ill, or respiratory-support populations. The cohort was strongly cesarean-predominant (88/111 with recorded delivery mode), and delivery mode can influence early lung-fluid clearance and respiratory adaptation. The small vaginal-delivery subgroup and absence of an adjusted analysis leave residual confounding by delivery mode. Gestational age within the term range, birth weight, sex, feeding status, behavioral/sleep state, maternal diabetes, labor exposure, and transient oxygen therapy may also influence measurements and were not incorporated into a multivariable longitudinal model.

Diaphragm ultrasound is operator-dependent. Follow-up was limited to 48 h, preventing assessment of later neonatal trajectories or stability beyond the immediate transition.

### 4.9. Future Directions

Future studies should include larger, multicenter cohorts and extend the observation period beyond the early neonatal phase.

In addition, integrating diaphragm ultrasound with clinical outcomes such as respiratory support requirement, feeding tolerance, and NICU admission could help define its prognostic value.

Reference intervals should be developed only after standardized acquisition, formal reproducibility testing, prespecified sample-size planning, balanced representation of delivery modes and relevant clinical strata, and external multicenter validation.

## 5. Conclusions

In this single-center cohort of 112 term neonates, LUS decreased substantially during the first 48 h after birth, whereas bilateral diaphragmatic excursion increased only modestly and DTF remained stable. The diaphragm effect sizes were small and their clinical significance is uncertain. These data provide preliminary descriptive trajectories, not clinical reference values. Multicenter studies with standardized timing, operator-reliability assessment, appropriate adjustment for delivery mode and other covariates, and longer follow-up are required before clinical implementation.

## Figures and Tables

**Table 1:** Baseline demographic and clinical characteristics of the analyzed cohort (*n* = 112).

Characteristic	Value
Male sex	60/112 (53.6%)
Female sex	52/112 (46.4%)
Gestational age, weeks	38.70 ± 1.12
Birth weight, g	3295.89 ± 473.01
Cesarean delivery	88/111 (79.3%); 1 missing
Vaginal delivery	23/111 (20.7%); 1 missing
Supplemental oxygen at 1 h	24/112 (21.4%)
Supplemental oxygen at 24 and 48 h	0/112 (0%)

**Table 2:** Serial ultrasound measurements at 1, 24, and 48 h of life.

Outcome	1 h	24 h	48 h	Omnibus Result
Right expiratory thickness, mm	2.25 ± 0.27 (2.20–2.30)	2.18 ± 0.25 (2.13–2.23)	2.26 ± 0.30 (2.20–2.32)	χ^2^ = 6.43; *p* = 0.040; W = 0.029
Right inspiratory thickness, mm	2.72 ± 0.28 (2.67–2.77)	2.66 ± 0.30 (2.60–2.72)	2.72 ± 0.35 (2.65–2.79)	*p* = 0.206
Left expiratory thickness, mm	2.24	2.2	2.24	*p* = 0.304
Left inspiratory thickness, mm	2.7	2.64	2.7	*p* = 0.497
Right excursion, mm	5.65 ± 0.71 (5.52–5.78)	5.80 ± 0.62 (5.68–5.92)	5.91 ± 0.58 (5.80–6.02)	χ^2^ = 7.47; *p* = 0.024; W = 0.033
Left excursion, mm	5.64 ± 0.69 (5.51–5.77)	5.80 ± 0.62 (5.68–5.92)	5.91 ± 0.54 (5.81–6.01)	χ^2^ = 6.83; *p* = 0.033; W = 0.031
Right DTF, %	20.96 ± 3.93 (20.22–21.70)	22.05 ± 4.99 (21.12–22.98)	20.93 ± 3.78 (20.22–21.64)	*p* = 0.065
Left DTF, %	21.52 ± 3.97 (20.78–22.26)	22.51 ± 4.75 (21.62–23.40)	21.72 ± 3.79 (21.01–22.43)	*p* = 0.252
Total LUS	7.01 ± 2.64 (6.52–7.50)	1.20 ± 1.46 (0.93–1.47)	0.09 ± 0.35 (0.02–0.16)	χ^2^ = 198.14; *p* < 0.001; W = 0.885

Data are mean ± SD (95% CI of the time-point mean), calculated with *n* = 112, where complete observations were assumed. DTF, diaphragmatic thickening fraction; LUS, lung ultrasound score; W, Kendall’s coefficient of concordance.

## Data Availability

The original contributions presented in this study are included in the article. Further inquiries can be directed to the corresponding author.

## Supplementary Materials

The following supporting information can be downloaded at: https://www.mdpi.com/article/10.3390/diagnostics16172733/s1, Figure S1. Schematic representation of the neonatal thoracoabdominal region showing the probe positions used for diaphragm ultrasound. (A) For diaphragmatic thickness and thickening fraction, a high-frequency linear transducer is placed on the lateral thoracic wall at the mid-axillary line, be-tween the eighth and tenth intercostal spaces, over the zone of apposition. The diaphragm is visualized in B-mode as a three-layer structure between the pleural and peritoneal interfaces. (B) For diaphragmatic excursion, a curvilinear transducer is positioned below the costal margin using a subcostal approach and directed toward the diaphragmatic dome. The M-mode cursor is aligned as perpendicular as possible to the direction of diaphragmatic movement.
