# Supplementary material for: Serial Ultrasound Assessment of Diaphragmatic Function in Term Neonates During the First 48 Hours of Life
Source: Diagnostics (Basel). 2026 Aug 26;16(17):2733. doi: 10.3390/diagnostics16172733 (PMC13565314; doi:10.3390/diagnostics16172733)
Supplement: Supplementary file 1 [file diagnostics-16-02733-s001.zip › diagnostics-4439984-supplementary.pdf]

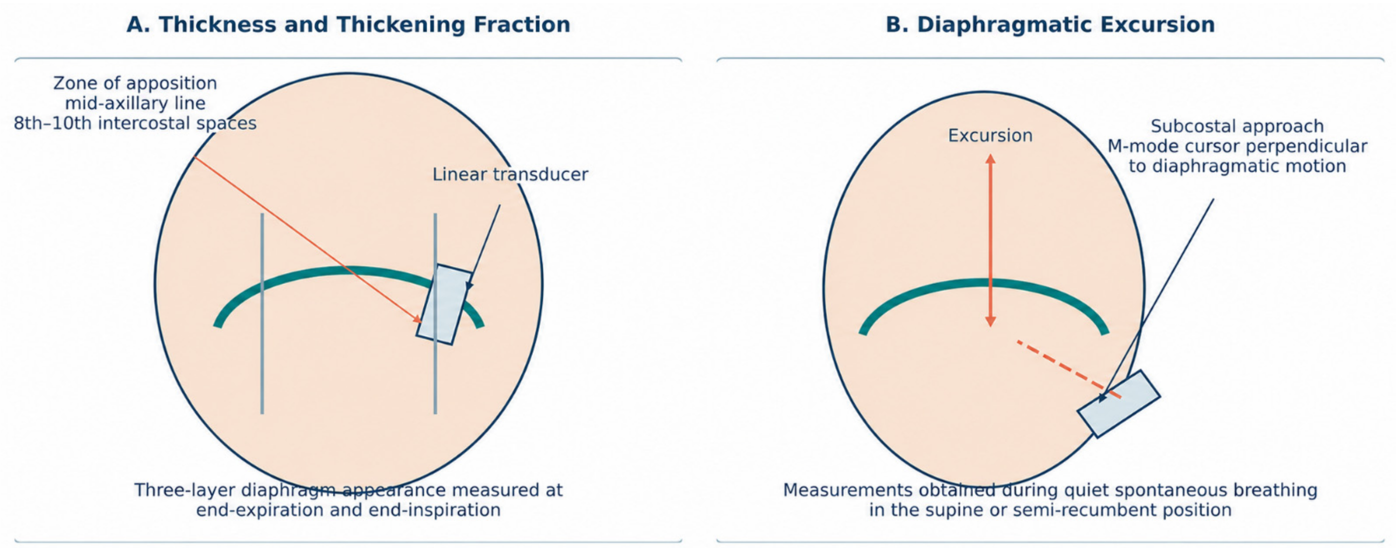

Figure S1. Schematic representation of the neonatal thoracoabdominal region showing the probe positions used for diaphragm ultrasound. (A) For diaphragmatic thickness and thickening fraction, a high-frequency linear transducer is placed on the lateral thoracic wall at the mid-axillary line, between the eighth and tenth intercostal spaces, over the zone of apposition. The diaphragm is visualized in B-mode as a three-layer structure between the pleural and peritoneal interfaces. (B) For diaphragmatic excursion, a curvilinear transducer is positioned below the costal margin using a subcostal approach and directed toward the diaphragmatic dome. The M-mode cursor is aligned as perpendicular as possible to the direction of diaphragmatic movement.
